# Developmental Biology and Induction of Phi Thickenings by Abiotic Stress in Roots of the Brassicaceae

**DOI:** 10.3390/plants7020047

**Published:** 2018-06-19

**Authors:** Maketalena Aleamotu’a, Yu-Ting Tai, David W. McCurdy, David A. Collings

**Affiliations:** School of Environmental and Life Sciences, The University of Newcastle, Callaghan, NSW 2308, Australia; maketalena.akauola@uon.edu.au (M.A.); yu-ting.tai@uon.edu.au (Y.-T.T.); david.mccurdy@newcastle.edu.au (D.W.M.)

**Keywords:** phi thickening, secondary wall, Brassicaceae roots, *Brassica oleracea*, *Brassica napus*, salt stress, water stress

## Abstract

Phi thickenings are specialized bands of secondary wall deposited around radial walls of root cortical cells. These structures have been reported in various species from the Brassicaceae, including *Brassica oleracea*, where previous reports using hydroponics indicated that they can be induced by exposure to salt. Using roots grown on agar plates, we show that both salt and sucrose can induce the formation of phi thickenings in a diverse range of species within the Brassicaceae. Within the genus *Brassica*, both *B. oleracea* and *B. napus* demonstrated the formation of phi thickenings, but in a strongly cultivar-specific manner. Confocal microscopy of phi thickenings showed that they form a complex network of reinforcement surrounding the inner root cortex, and that a delicate, reticulate network of secondary wall deposition can also variously form on the inner face of the cortical cell layer with phi thickenings adjacent to the endodermal layer. Results presented here indicate that phi thickenings can be induced in response to salt and water stress and that wide variation occurs in these responses even within the same species.

## 1. Introduction

Phi thickenings are peculiar secondary cell wall structures found in the cortex of plant roots in diverse species ranging from gymnosperms to angiosperms and from monocots to eudicots. They were first reported in transverse sections in the ring of cells surrounding the outside of the endodermis in roots of *Taxus baccata* by Van Tieghem [1], who described the cell wall thickenings with the French term “reseau sus-endodermique” which might be translated to “super-endodermal” or “peri-endodermal” network. Observations of the roots of several Rosaceae species by Russow in 1875 showed similar coordinated wall thickenings in adjacent cortical cells that resembled the Greek letter Φ (phi), and this lead to the adoption of the name “phi thickening” [2] to describe localised wall thickenings seen in cortical cells of roots. While similar structures have now been observed in many species, we broaden the definition of phi thickenings to include specialised, reticulate, or localised band-like secondary thickenings that form only around the cell wall of root cortical cells, rather than across the entire wall surface. Thus, our definition would also include the more complex wall thickenings in the roots of some epiphytic orchids, a series of structures sometimes referred to as a ‘pseudovelamen’ [3,4]. Another related type of cell wall thickening in the root cortex has been described as a ‘crescent thickening’ because the thickening is limited to the inner faces and sides of the cortical cells [5,6]. These findings confirm that multiple types of secondary cell wall thickenings are present in the cortical cells of plant roots and would argue for our more comprehensive definition of phi thickenings. 

In the early literature [7], phi thickenings were classified into three types based on the location of phi cell layers (the layer of root cortex in which the phi thickenings are present). A phi cell layer located in the innermost layer of cortex adjacent to the endodermis is defined as Type I, and it was this organisation that Van Tieghem described as “sus-endodermique”. Type II thickenings occur when the phi cell layer is located in the outermost layer of the cortex adjacent to the epidermis, an organisation described by Van Tieghem as “sous-épidermique” or sub-epidermal, while Type III defines phi cell layers in the intermediate cortex, either in single or multiple layers. Van Tieghem [7] observed that it is not common to have more than one type of phi thickening in the same root. This classification is still used to differentiate the various locations of phi thickenings in roots. 

Although phi thickenings were first described in the 19th century, relatively little is known about the function(s) they might perform in plant roots. One early suggestion for the role of phi thickenings was that they might play a similar role to the Casparian strip by regulating solute uptake [8]. Both phi thickenings and Casparian strips are cell wall thickenings reinforced with impermeable lignin polymers, and they are found in root cortical cells of some species and endodermal cells of most species, respectively. Casparian strips are typically impregnated with the wax suberin whereas phi thickenings contain little if any suberin [9,10]. The lack of suberin in phi thickenings need not mean that these structures do not function in the regulation of transport regulation, as suberin-free mutants in *Arabidopsis* retain a functional Casparian strip that can block the movement of apoplastic tracer dyes [11]. However, similar dye uptake studies in both apple and geranium roots showed that dye movement was blocked by the endodermis but not the phi thickenings [12]. Another proposed function of phi thickenings is that they provide mechanical support for the root cortex, and that they may act as reinforcing structures for the root cortex [13]. Interestingly, Melville, et al. [14] proposed that they might act as a physical barrier against penetration by fungal hyphae. A more recent study suggested that phi cells may play a part in active uptake of cations and anions for accumulation in vacuoles [15]. All these suggestions, however, have not been tested in detail, and it remains entirely feasible that phi thickenings perform multiple and different roles in roots of different species. 

Phi thickenings have been identified in a wide variety of plant species, but no systematic search for the presence of phi thickenings within a given plant family has been conducted since a series of studies by Van Teighem in the 1880s. Consequently, our current study provides a survey of phi thickening development in the Brassicaceae and also assesses the response of the species to salt-induced water stress. We selected this family because 19th century reports suggest that phi thickenings are widespread within the Brassicaceae [16,17] (although they do not, apparently, occur in *Arabidopsis thaliana*) and because phi thickenings can be induced in *Brassica oleracea* roots by 14-d hydroponic treatment with salt [15,18]. By analysis of 5-d-old seedlings grown on nutrient agar plates in the presence or absence of added salt, we document considerable variation within the Brassicaceae in not only the presence of phi thickenings but also their response to salt stress. However, induction also occurred under sucrose-induced water stress in conditions that did not inhibit root elongation, thus demonstrating that root growth inhibition and phi thickening induction are not directly linked. We also report variations in the ability to form phi thickenings within two species, *B. oleracea* and *B. napus*, where only some cultivars induce phi thickenings strongly in response to salt. Using confocal microscopy and 3D reconstructions, we also show that phi thickenings form a continuous, lignified ring around the inner cortex of some *Brassica* roots, immediately outside the endodermis, and more intriguingly, that these thickenings often extend to form a delicate, reticulate network of secondary wall material along the inner face of these cells adjacent to the endodermal layer. Collectively, these observations reveal the diverse nature of phi thickening development in the Brassicaceae, and that variations can occur in phi thickening responses to abiotic stress not just within a family but even within a single species. 

## 2. Results

### 2.1. Phi Thickenings Response to Varying Salt and Sucrose Concentrations

**Figure 1:**
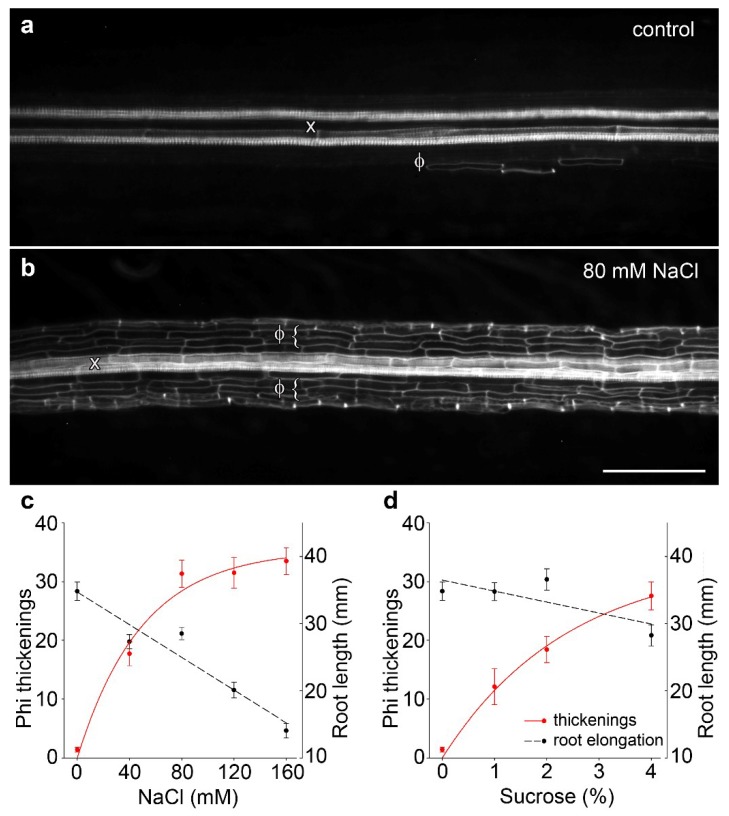
Water stress-induced phi thickenings in *B. oleracea* cultivar ‘Marathon F1’. (**a**) Control root grown for 4 d then cleared and stained with basic fuchsin and viewed using a fluorescence dissecting microscope. Longitudinally mounted roots show heavily-stained xylem vessels (x) and rare phi thickenings (Φ); (**b**) Root grown for 4 d in presence of 80 mM NaCl then processed as described for control roots. Extensive deposition of phi thickenings (Φ) is seen surrounding the central stele and associated xylem (x). Bar = 200 μm; (**c**,**d**) Phi thickening induction in response to NaCl (**c**) or sucrose (**d**). Seedlings were germinated and grown for 4 d in the presence of varying concentrations of NaCl or sucrose. Scoring involved counting induced cells within a set length of root (left axis, dotted lines) and was not related to root elongation (right axis, solid red lines). Data are means ± SEM, *n* > 21 plants for each data point.

Following previous work in *B. oleracea* in which 80 mM salt treatment administered through hydroponics induced phi thickenings [15,18], we confirmed that salt treatment can induce the formation of phi thickenings in cultivar ‘Marathon F1’. In our experiments, we replaced hydroponics [15] with growth of seedlings for 4 d on agar plates containing 1/2 MS salts or 1/2 MS salts supplemented with 80 mM NaCl. Whole roots were fixed, cleared with potassium hydroxide, and stained for lignin using basic fuchsin [16,19]. Compared to salt-free controls, which only rarely contained phi thickenings (Figure 1a), 80 mM NaCl induced strong development of phi thickenings immediately outside the central stele (Figure 1b). Using images derived from a fluorescence dissecting microscope, we developed a scoring system based on counts of the number of induced phi cells (i.e., cortical cells containing phi thickenings) within a set length of cleared root. We used this system to score the response of phi thickenings to varying salt (Figure 1c) and sucrose concentrations (Figure 1d). The response curve to salt stress emphasized that the degree of phi thickening formation increased with salt concentration, and this increase was accompanied by reduced root elongation (Figure 1c). Importantly, induction of phi thickenings was not specifically a response to salt stress, since induction was also seen in response to sucrose (Figure 1d). Furthermore, whereas salt-induced phi thickenings increased as root elongation decreased, this response was not seen with sucrose, where root elongation was not significantly different at an intermediate sucrose concentration (2%) at which strong induction of phi thickenings occurred. Therefore, we conclude from this analysis that root elongation is not correlated with phi thickening formation, which suggests that phi thickenings play a role other than growth in roots.

### 2.2. Survey of Phi Thickening Development in the Brassicaceae 

We extended the survey of phi thickening formation in different species from the Brassicaceae conducted by Van Tieghem [16] by investigating the induction of phi thickenings by salt. We also surveyed the salt-induction of phi thickenings in different cultivars of both *B. oleracea* and *B. napus*. Within the seven major groups of *B. oleracea*, and within the 20 cultivars tested, all except one contained phi thickenings to some extent (Table 1). The cultivars in which phi thickenings were abundantly induced were mainly the Italica group of *B. oleracea* (broccoli), whereas the Gongylodes and Botrytis groups (kohlrabi and cauliflower, respectively) typically had fewer phi thickenings, and with the exception of Romanesco, were not strongly induced by salt. We also measured root length of the seedlings and determined how the inclusion of 80 mM NaCl affected root growth. As shown by the root length ratio (Table 1), some cultivars were more resistant to the effects of salt than others. 

Within other species of *Brassica*, similar variations in phi thickening induction were also observed (Table 2). Within the five canola cultivars of *B. napus* surveyed, major variation was seen, whereby the three spring cultivars ‘Hyola97CL’, Canola ‘Hyola474CL’, and Canola ‘Archer’ showed strongly-induced phi thickenings, whereas the two winter canola cultivars tested, ‘Edimax’ and ‘Sensation’, either mildly or completely lacked induced phi thickenings, respectively. In other *Brassica* species, however, with the exception of occasional thickenings present in the *B. rapa* subspecies Pekinesis ‘Wong bok’, no substantial induction was observed (Table 2). In conclusion, the induction of phi thickenings in the different *Brassica* species was shown to be strongly dependent on species and cultivar. 

We also surveyed phi thickenings and salt induction within other genera of the Brassicaceae. Contrary to previous observations [16], we found that phi thickenings were generally rare in other genera, and they were not typically induced by salt treatments (Table 3). Extensive phi thickenings were seen in *Sinapsis alba* (white mustard) in both salt-free and 80 mM NaCl conditions, with the higher phi cell scores attributed to the comparatively short length of cells present within the inner cortex of this species, which provided more cells within the defined measurement length. *Thlaspi caerulescens* (alpine pennycress) also showed high levels of phi thickening induction in both salt-free and 80 mM NaCl conditions. Other species in which phi thickenings were observed included *Iberis amara* (candytuft) and *Lobularia maritima* (alyssum) that showed strong, salt-induced formation of phi thickenings equivalent to those of the *Brassica* species. 

### 2.3. Structure and Characteristics of Phi Thickenings

We characterised the structure of phi thickenings by confocal microscopy. Lignification of the phi thickenings, Casparian strip, and xylem could be viewed in whole, cleared roots with conventional lignin stains viewed under bright field, or by imaging blue, lignin autofluorescence from unstained samples resulting from 405 nm (violet) excitation by confocal microscopy (data not shown). However, for high-resolution confocal imaging, cleared roots were double-stained with berberine hemisulfate for lignin (and suberin) [3,4,20,21,22] and for cellulose with pontamine fast scarlet 4B [23,24]. This combination of stains allowed comparison of lignified structures with the location of non-lignified cell walls. All confocal images were taken at approximately the middle section of the root between the root tip and hypocotyl where phi thickenings were typically most intensely induced. 

**Figure 2:**
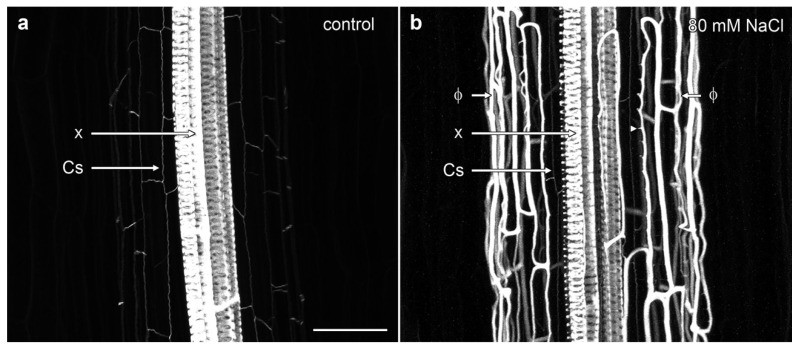
Maximum projection confocal optical stacks of berberine-stained lignin in *B. oleracea* roots of cultivar ‘Marathon F1’. (**a**) Control root in the absence of added salt. The Casparian strip (Cs) was weakly labelled while the xylem bundles (x) were strongly labelled; (**b**) Root grown in presence of 80 mM NaCl, which strongly induced the formation of phi thickenings (Φ). Bar = 100 μm.

Confocal imaging demonstrated the complex nature of phi thickenings in the cortex and the more weakly labelled Casparian strip within the endodermis, with both structures surrounding the heavily lignified xylem vessels in the central stele. In non-induced *B. oleracea* cv ‘Marathon F1’ roots, only the Casparian strip was visible as wavy, thin lines outlining the shape of the narrow endodermal cells (Figure 2a, Supplementary Movie S1). However, in the presence of salt, a strongly lignified network of net-like bands of phi thickenings was induced in the inner cortical cells. This network almost completely masked the labelling from the weakly lignified Casparian strip surrounding the vascular bundle (Figure 2b). Three dimensional (3D) reconstructions emphasised the unusual architectural organisation of phi thickenings as they extended along the *B. oleracea* root (Supplementary Movie S2). One unusual aspect was the ladder-like network of wall thickenings present on the inner face of the inner cortical cell wall, immediately adjacent to the endodermis (Figure 2b, arrowshead). These structures will be discussed in more detail in the next section. 

**Figure 3:**
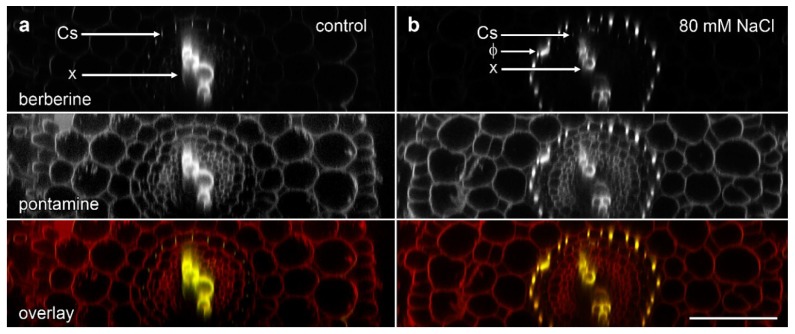
Concurrent confocal images of pontamine-stained cellulose (red in overlay) and berberine-stained lignin (green in overlay, appearing as yellow when colocalised with pontamine-stained cellulose) in *B. oleracea* ‘Marathon F1’ roots. Images are computer-generated re-slices through optical stacks collected from roots mounted longitudinally. (**a**) Control root showing absence of phi thickenings; (**b**) Root grown for 4 d in the presence of 80 mM NaCl which induced formation of phi thickenings (Φ) in the innermost cortex immediately outside the endodermis. In both roots, Casparian strips (Cs) in the endodermis are weakly labelled with berberine while xylem (x) strongly labelled in the central vascular tissue. Bar = 100 μm.

We used the re-slice function in ImageJ to convert the data sets of roots imaged in longitudinal view into cross-section (Figure 3). We imaged ‘Marathon F1’ and confirmed that, similar to previous studies [15,18], lignified phi thickenings were induced by salt in cells in the innermost layer of the cortex. These cells were immediately outside the endodermis in which berberine staining showed a faint signal for the weakly lignified Casparian strip (Figure 3b). This pattern, also visible with pontamine-labelled cellulose, represents Type I phi thickening [9,10]. The pontamine counterstaining also confirmed that this layer of phi thickening-containing cells was the innermost of the three layers of cortical cells, and that these cells were smaller than the outer two (or sometime three) cortical layers (Figure 3b). In control plants in which no induction had occurred (Figure 3a), the Casparian was again faintly visible, but there was no indication of the presence of the phi thickenings, either with berberine staining of lignin or pontamine staining of cellulose.

Similar phi thickening induction was observed in 4 d-old seedlings of *B. napus* in a spring canola cultivar, ‘Hyola971CL’, when grown on nutrient agar plates containing salt. In ImageJ-derived cross-sections, phi thickening development was absent in control roots but induced in the innermost of three cortical cell layers in patterns similar to that seen in *B. oleracea* (Figure S1). 

**Figure 4:**
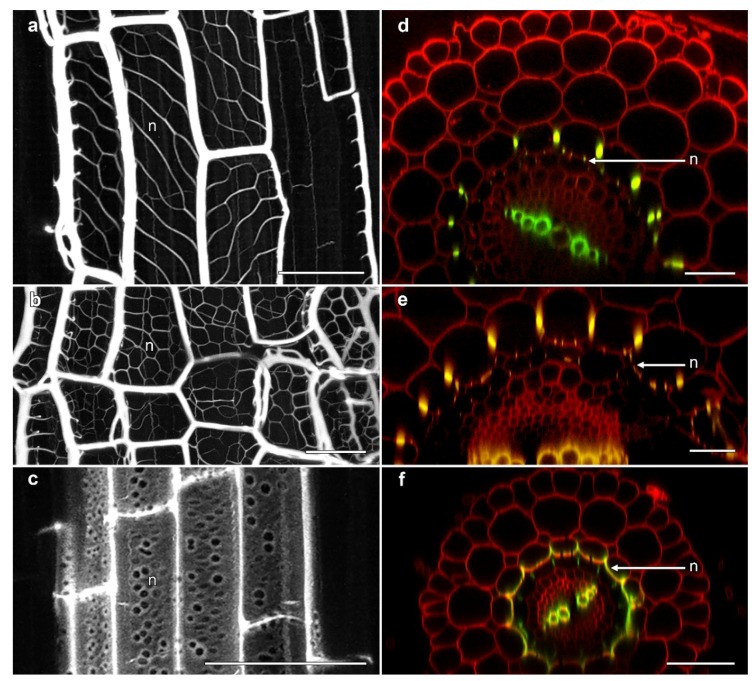
Confocal imaging of fine reticulate networks associated with phi thickenings in Brassicaceae roots. (**a**–**c**) Average projections of longitudinal sections showing lignin stained with berberine; (**d**–**f**) Computer-generated re-slices through optical stacks collected from roots mounted longitudinally showing berberine-stained lignin (green) and pontamine-stained cellulose (red); (**a**,**d**) *B. napus* ‘Hyda971CL’ 5 day-old root treated with 80 mM NaCl induced the formation of phi thickenings in the innermost cortex and reticulate network (n) at the inner face of the innermost cortical layer adjacent to the Casparian strip-containing endodermis; (**b**,**e**) *S. alba* ‘White mustard’ 5 day-old root treated with 1% sucrose induced the formation of phi thickenings in the innermost cortex and reticulate network (n) at the inner face adjacent to the endodermis; (**c**,**f**) *T. cauelescens* 8 day-old root treated with 1% sucrose induced the formation of lignified thickenings along the inner face of the inner cortex adjacent to the endodermis. All bars = 50 μm.

Several unusual aspects of phi thickening development were observed by confocal microscopy. The most notable of these was the development of regular, lignified thickenings on the inner face of the inner cortical cells along the wall immediately adjacent to the endodermis, although variation in the architecture of these structures were observed across the Brassicaceae. In *B. napus* ‘Hyda971CL’, a network of delicate ladder-like thickenings were observed that were organised at non-transverse angles (Figure 4a). In *Sinapsis alba* (white mustard), however, the angle of these structures was slightly different, thus forming a more uniform net-like structure which was extensively lignified (Figure 4b). The net-like structures in ‘Marathon F1’ and ‘Red cabbage ruby ball F1’ (data not shown) were similar to networks seen in *S. alba* (white mustard). In cross-section, these delicate wall thickenings were visible as small, lignified specks on the inner-face of the inner cortical cells (Figure 4d,e). Distinct from the reticulate structures observed in *Brassica* and *Sinapsis*, extensions from the phi thickenings were seen along the cell wall between the inner cortex and the endodermis in salt-treated roots of *T. caerulescens*. Here, the lignified phi thickening extended all the way along the inner face of the inner cortical cells (Figure 4f), so that in cross-section these structures appeared more like “crescent thickenings”, as described in roots of several fruit trees [5,6], rather than typical phi thickenings. In longitudinal view, the walls displayed the formation of pore-like structures between the inner cortex and the endodermis (Figure 4c). Similar, lower-resolution images were previously shown for heavy metal-induced phi thickenings in *T. caerulescens* roots [25], and it is possible that structures in *Thlaspi* roots previously described as “a second layer of endodermis cells” may also reflect this development of the phi thickening layer in the inner cortex [26]. 

These reticulate extensions of the phi thickening network from the radial walls of the inner cortical cells to the inner face of the cells were first reported by Woronin in 1878 [17] and Van Tieghem in 1887 [16]. However, these are the first actual images of their appearance in both longitudinal and cross-section to be published. These structures would, however, be the “wall ingrowths” reported in electron micrographs of salt-treated *B. oleracea* roots [15]. 

### 2.4. Phi Thickenings in Brassica Oleracea Adventitious Aerial Roots

**Figure 5:**
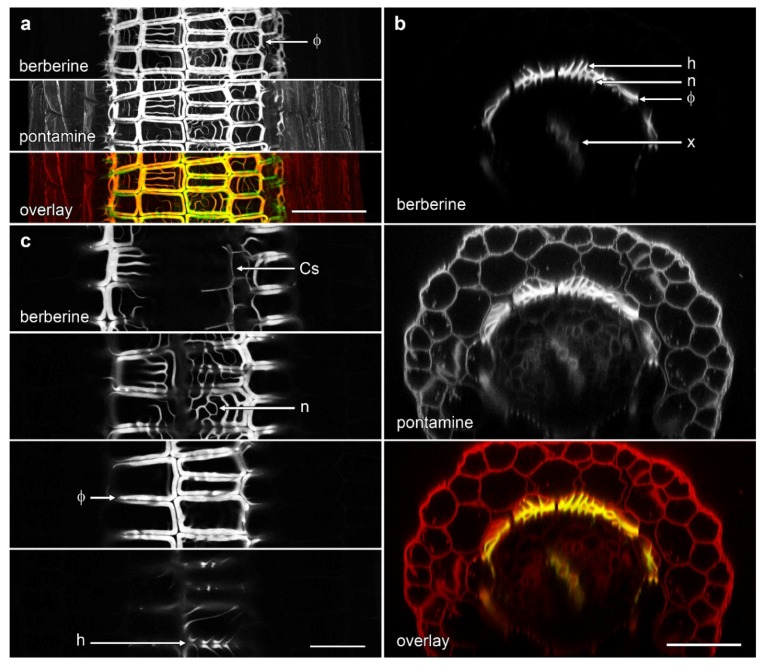
Phi thickenings in adventitious aerial roots of *B. oleracea* ‘Marathon F1’. (**a**) Confocal three dimensional (3D) reconstructions of longitudinal sections stained with berberine (lignin, green in overlay) and pontamine (cellulose, red in overlay) containing extensive formation of phi thickenings (Φ). Bar = 100 μm; (**b**) Computer-generated re-slices through optical stacks of longitudinal sections showing phi thickenings (Φ) at the inner cortex, reticulate network (n) at the inner face of the inner cortex, and hook-like structure (h) projecting outwards from the radial bands of the phi thickenings. Bar = 100 μm; (**c**) Projections of 2 µm of optical sections showing berberine-stained lignified cell walls in different focal planes demonstrating the Casparian strip (Cs), reticulate network (n), phi thickenings (Φ), and hook-like structure (h). Bar = 50 μm.

Extensive formation of phi thickenings was identified in aerial roots developing from stem nodes of *B. oleracea* cv ‘Marathon F1’ (Figure 5). These roots were relatively short and clustered together and, unlike primary roots, had only two layers of cortical cells. Phi thickenings were present in the innermost cortical cell layer as thick bands that separated cells from one another (Figure 5a,b). A heavily-lignified reticulate network of thickenings with irregular patterning also formed at the inner face of the inner cortex adjacent to the endodermis containing Casparian strip (Figure 5b,c). This network could also project outwards as hook-like structures from the radial bands of the phi thickenings towards the outer wall of the inner cortical layer (Figure 5b,c).

## 3. Discussion

The classical definition of phi thickenings, that of paired thickenings within the radial cell walls in the root cortex arranged to form a band around the cell, is based on early observations of Russow, Woronin, and Van Tieghem [1,2,7,16,17]. We expand the definition of phi thickenings here to formally include all examples of secondary wall thickenings within the root cortex that are localised to specific regions of the wall. With this expanded definition, we can include the complex reticulate network present on the inner face of the inner cortical cells, the crescent thickening-like arrangement of the network in *Thlaspi* [25] that is similar to the crescent thickenings found in some fruit trees [5,6], and the more complex thickenings present in the aerial roots of many epiphytic orchids [3,4,20,21]. 

### 3.1. Phi Thickening Induction by Salt- and Sucrose-Induced Water Stress

The lignified secondary bands of phi thickenings observed in the primary roots of *Brassica* are typical Type I thickenings located in the innermost layer of the cortex adjacent to the endodermis. Similar type I phi thickenings were found to be induced by salt in lateral roots (data not shown), and the more dramatic thickenings observed in aerial roots of *B. oleracea* were also type I thickenings. Only a few studies have been conducted which record phi thickenings in the Brassicaceae [15,17,18,25,27,28], with the only previous taxonomic survey being that of Van Tieghem’s report from the 19th century [16]. This observation is surprising given that some members of the Brassicaceae are important crop species, and that phi thickenings can be induced in *B. oleracea* by salt stress [15,18].

In the last two decades, experiments have demonstrated that phi thickenings can be induced in cells in response to various environmental stresses including heavy metals, soil compaction, salt, water, and freezing stress [9,15,29,30,31,32]. Soukup, et al. [33] demonstrated that the formation of phi thickenings in *Prunus avium* L. are potentially environmentally controlled because they can only be induced ex vitro but not in vitro. These studies have suggested that the induction of phi thickenings may mechanically support the cortical cells, although the role(s) played by phi thickenings, and why such a wide variety of plants develop them, have so far remained unknown. Our results extend the observations of phi thickenings and demonstrate that these structures can be induced by both salt- and sucrose-induced water stress. More importantly, we also described an abundant reticulate network in aerial roots of 5 month-old *B. oleracea* cv ‘Marathon F1’ plants. Since aerial roots are prone to water stress and exhibit many other water stress-related structural changes [34], this result may provide further insight into the role played by phi thickenings in response to water stress. Aerial roots of epiphytic orchids also contain extensive phi thickenings [3,4,20,21], which can be readily induced by water stress in the orchid *Miltoniopsis* [35]. Furthermore, the fact that root elongation in *Brassica* is not directly linked with the induction of phi thickening formation may indicate that phi thickenings play a role in root biology other than regulating root growth. 

### 3.2. A Survey of Phi Thickenings in the Brassicaceae 

Since phi thickenings are present in a wide diversity of plants from angiosperms to gymnosperms, the ability to generate phi thickenings has either evolved multiple times during evolution or has been conserved for a very long period of time. In either case, the wide occurrence of phi thickenings indicates a fundamental importance for root development and function. 

No systematic search for the presence of phi thickenings within a given family has been conducted since the 1880s. We expanded on the most recent survey of the Brassicaceae by Van Tieghem in 1887 [16] by also analysing the response of roots to salt treatment. Our survey of the presence of phi thickenings and their induction by salt revealed wide variation within the Brassicaceae. Some of our observations were contrary to findings of Van Tieghem, who reported phi thickenings in some species such as *Brassica carinata*, *B. nigra*, *Cheiranthus cheiri*, *Lepidium sativum*, *Isatis* sp. *Crambe* sp., and *Malcolmia* sp., whereas we observed that these species lacked phi thickenings, and phi thickenings were not induced by salt. It is possible that the undescribed growth conditions used by Van Tieghem induced thickenings either under control or salt-induced conditions. Further, our observations have been limited solely to the young primary root. However, as noted by Van Tieghem, the thickening network develops at different ages in different genera. While he noted that the network developed in young primary roots in *Sinapsis*, in other genera such as *Raphanus* (radish) and *Malcolmia*, the formation of thickenings occurs only shortly before the start of secondary growth of the root. It is also possible that differences in species and cultivars resulted in our different observations. In particular, the diversity in the appearance and induction response within the different cultivated species of the genus *Brassica* represent only a small portion of the vast variability that occurs in this genus. It is possible that gene(s) responsible for regulating the induction of phi thickenings could have been mutated or lost in some cultivars during domestication and breeding.

To date, there is no explanation as to why only some species in the Brassicaceae have the ability to form phi thickenings while others apparently do not. However, in the case of the well-characterised species *Arabidopsis thaliana*, which has not been reported to form phi thickenings, it is possible that structural reasons account for a loss in the ability to form such thickenings. In this context, the number of cortical cells formed within the primary root varies substantially within the Brassicaceae. From our confocal reconstructions, *Brassica* species which form phi thickenings in primary roots have three (or four) layers, *Sinapsis* has three layers and *Thlaspi* has two, while in *Brassica* aerial roots, only two layers are seen. In all cases, however, cells of the inner cortical layer are smaller than the outer cortical layers. In contrast, the *Arabidopsis* primary root contains only a single layer of cortical cells, and structurally these appear similar to the outer cortical cells of *Brassica* and other Brassicaceae. Therefore, the loss of the inner cortex in *Arabidopsis* roots may explain the absence of phi thickenings in this species. However, since different cultivars of *Brassica* lack phi thickenings and differences are seen in root structure of the hyperaccumulator *Thlaspi caerulenscens*, which is capable of inducing phi thickenings, and the closely-related non-hyperaccumulator *T. arvense*, which does not [25], differences in root structure alone may not necessarily account for the presence/absence of phi thickenings. Therefore, root architecture may not be the sole reason why *Arabidopsis* does not appear to form phi thickenings. 

### 3.3. Phi Thickening Architecture 

To better understand the characteristics of phi thickenings, several studies have investigated their ultrastructure [15,36], anatomical development [21,32,37], and structural changes during primary and secondary growth [13]. Through the use of confocal microscopy and 3D reconstructions, we have provided more detailed analysis of their overall architecture in Brassicaceae roots. In traditional transverse section views of phi thickenings, the inner cortical cells where phi thickenings are present can be easily identified by the localised thickening between two adjacent cortical cells forming continuously around the inner cortical cells immediately outside the endodermis. However, when the cortical cells are viewed longitudinally, the lignified bands of phi thickenings can be seen as a network of rectangular frames around the inner cortical cells, which together appear as an elongated mesh wrapping around the central vascular bundle. 

More interestingly, confocal microscopy and 3D reconstructions also confirmed the presence of a delicate, reticulate network of secondary wall thickenings or ridges along the inner face of the inner cortical cells adjacent to the endodermis. The peculiarity and beauty of this structure was reported by both Woronin [17] and Van Tieghem [16], but such thickenings have not subsequently been described in the literature. Electron micrographs of induced *B. oleracea* roots showed small wall thickenings on the inner face of the inner cortical cells, which were described as “cell wall ingrowths” [15], but in light of our observations, probably represent this reticulate network seen in cross section. Questions arising then are how does the reticulate network form and how is it limited to the space between the inner cortex and the endodermis? Cortical microtubules run lengthwise along phi thickenings in roots of *Pelargonium* [36] and *Miltoniopsis* [21], and thus may have roles in directing reticulate network formation similar to that seen in secondary wall deposition [38]. 

A reticulate pattern was not present in *T. caerulenscens* where, in contrast, the inner wall of the inner cortical cells was more uniformly thickened. Together with the regular phi thickenings, this structure appeared similar to crescent thickenings in the root cortex of red bayberry (*Myrica rubra*) and wax apple (*Syzygium samarangense*) [5,6]. Work by Fernandez-Garcia, Lopez-Perez, Hernandez, and Olmos [15] in *B. oleracea* showed high ATPase activity localised to so-called wall ingrowth regions, an area equivalent to where reticulate networks are located, while Zelko, Lux, and Czibula [25] indicated that the increased surface area in the intercellular space adhering to the cortical thickenings may play a similar role to transfer cells [39] by increasing plasma membrane surface for enhanced transport capacity. However, these “wall ingrowth” structures are highly lignified and thus structurally different from the non-lignified, primary wall-like wall ingrowths which characterise most transfer cells [38,40]. Further, the interface between the inner cortex and endodermis would seem an unlikely place for a significant apoplasmic barrier, as trans-endodermal transport is mediated by symplasmic flow from the cortex to the endodermis.

## 4. Materials and Methods 

### 4.1. Plant Material

Seeds of various Brassicaceae species were purchased from commercial seed supply companies (Table 1, Table 2 and Table 3) with the exceptions of *Cardamine hirsuta*, *Thlaspsi caerulescens*, *B. napus*, and *Arabidopsis thaliana*, which were obtained from Prof John Bowman (Monash University, Melbourne, VIC, Australia), Dr Damien Callahan (Deakin University, Melbourne, VIC, Australia), Dr Susan Sprague (CSIRO Agriculture, Canberra, ACT, Australia), and Arabidopsis Biological Resource Center, respectively. Seeds were surface sterilised in 50% (*v*/*v*) ethanol and 3% (*v*/*v*) hydrogen peroxide for 2 min and rinsed extensively with sterile deionised water. Seeds were germinated on agar plates containing half-strength MS salts (Sigma-Aldrich, St Louis, MO, USA) and 1.2% (*w*/*v*) agar (Becton-Dickinson, East Rutherford, NJ, USA), sometimes supplemented with NaCl and/or sucrose to the stated concentrations. Plated seeds were stratified (4 °C, 2 d) and then grown with the plates positioned vertically in a growth cabinet (photoperiod of 16 h/8 h, temperature 22 °C/18 °C day/night, light ~120 μmol⋅m^−2^ s^−1^).

### 4.2. Fluorescence Staining 

Plants were removed from the growth cabinet at defined times and scanned for root length measurements using an Epson flat-bed scanner (300 dpi) using transmitted light. The seedlings were then fixed for at least 3 h in PME solution (50 mM PIPES, pH 7.2 (KOH), 2 mM MgSO_4_, and 2 mM EGTA) containing 3.7% (*w*/*v*) formaldehyde and 0.1% (*v*/*v*) dimethyl sulfoxide. The fixed seedlings were then washed several times in phosphate-buffered saline (PBS; 131 mM NaCl, 5.1 mM Na_2_HPO_4_, 1.56 mM KH_2_PO_4_, pH 7.2), and the roots cleared in 10% (*w*/*v*) KOH at 60 °C for at least 2 days to remove autofluorescent compounds from the tissue. Roots were rinsed several times in distilled water before fluorescence staining for lignin using either berberine hemisulfate (0.1% (*w*/*v*) in distilled water) for 5 min or basic fuchsin (0.001% (*w*/*v*) in distilled water) for 2 min, and for cellulose using pontamine fast scarlet 4B (0.1% (*w*/*v*) in 150 mM NaCl) for 5 min. All three stains were purchased from Sigma-Aldrich. Stained root tissues were mounted in 100% (*v*/*v*) glycerol using spacers to ensure that coverslips did not crush the roots. 

### 4.3. Microscopy

For phi thickening survey and induction experiments, whole roots stained with basic fuchsin were imaged using a Leica (model MZ FLIII) stereofluorescence dissecting microscope with 10× magnification and using green excitation. All images were taken using a Zeiss AxioCam HRc camera, processed using Zeiss Zen software, and they were then imported into ImageJ (National Institute of Health, Bethesda, MA, USA) for viewing and subsequent analysis. Scoring of phi thickening from dissecting microscope images was carried out by counting phi cells in one half of the root with most thickenings. Scoring of phi cells was performed in the middle section (lengthwise) of the root, between 0.5 and 3.5 cm from the root tip, and approximately half way along the root of the germinated seedling. 

For confocal microscopy, whole roots that were double-stained with berberine hemisulfate and pontamine fast scarlet 4B were mounted in 100% glycerol in a Fluorodish (World Precision Instruments, Sarasota, FL, USA). The roots were imaged using an Olympus FV1000 confocal microscope, using a 30× NA1.05 silicone oil immersion lens on an inverted microscope. Berberine (excitation 473 nm, emission 485–545 nm) and pontamine (excitation 559 nm, emission 570–670 nm) were recorded in sequential mode along with transmitted light images in DIC mode. Confocal optical sections were collected through the entire root at 0.5 µm intervals. These high-resolution stacks were collected with the pinhole minimised to improve Z-resolution, and to optimise cross-sections generated in ImageJ using the re-slice function. ImageJ was also used to generate projections and rotations of the data. 

## 5. Conclusions

Our survey of phi thickening development and response to salt and water stress in the Brassicaceae has revealed substantial variation across the family and even within different cultivars of the same species. The strong cultivar-dependent variations in the induction of phi thickenings by salt or water stress will enable experimentation to directly test the function(s) of phi thickenings in roots, including possible roles in the regulation of solute uptake, and in providing mechanical strength to the root. In such experiments, cultivars lacking induction could be thought of as phi thickening-deficient mutants. Dissection of the genetic pathways involved in the regulation of phi thickening development might also benefit from comparisons between cultivars. Such information will be directly applicable to crop breeding strategies in *Brassica*, and other agricultural crops where phi thickenings occur, to assist in the development of crops with improved resistance to salt and water stress.

## Figures and Tables

**Table 1:** Phi thickening induction by salt in different *Brassica oleracea* groups and cultivars.

Group	Cultivar	Source Code	Measurement (Days)	Root Length (cm)		Root Length Ratio	Phi Thickening Score	
				0 mM	80 mM	(80 mM/0 mM)	0 mM	80 mM
Acephala kale, collard greens	‘Dwarf green curled’	1	10	1.9 ± 0.3	2.2 ± 0.6	1.09	0	19
Alboglabra Chinese broccoli	‘Kailaan’	2	5	3.2 ± 0.6	3.4 ± 0.5	1.06	8	21
Gemmifera Brussels sprouts	‘Evesham special’	1	10	4.9 ± 1.2	8.5 ± 0.1	1.73	21	25
	‘Long Island improved’	3	6	3.4 ± 0.3	3.0	0.86	0	10
Gongylodes kohlrabi	‘White Vienna’	4	13	2.4 ± 1.2	1.5 ± 0.5	0.62	0	6
	‘Purple Vienna’	4	5	3.7 ± 0.8	2.2 ± 0.4	0.59	0	1
Capitata cabbage	‘Mammoth red rock’	4	5	4.9 ± 1	4.6 ± 0.8	0.94	3	45
	‘Golden acre’	4	6	4.3 ± 0.4	3.5 ± 0.5	0.80	0	2
	‘Ruby ball F1’	1	5	5.1 ± 0.4	4.6 ± 0.2	0.90	18	43
Botrytis cauliflower, broccoflower, Romanesco broccoli	‘Quickheart’	1	5	4.4 ± 0.2	3.1 ± 0.4	0.70	1	15
	‘Phenomenal early’	2	5	4.9 ± 0.4	3.1 ± 0.4	0.64	3	0
	‘Snowball’	2	11	1.0 ± 0.2	1.0 ± 0.2	0.99	14	15
	‘Romanesco’	3	6	4.3	3.0 ± 1.5	0.69	4	47
Italica broccoli	‘Marathon F1’	1	5	4.8 ± 0.6	4.1 ± 0.2	0.86	21	39
	‘Calabrese’	3	6	3.3 ± 0.8	4.1 ± 0.02	1.25	33	43
	‘Di Ciccio’	3	11	2.3 ± 1.1	0.9 ± 0.1	0.40	8	20
	‘Belstar’	3	6	3.4 ± 0.4	2.7 ± 0.5	0.79	18	40
	‘Umpqua’	5	5	2.9 ± 0.7	3.2 ± 1.0	1.10	22	39
	‘Goliath’	5	5	4.4 ± 0.7	4.2 ± 0.3	0.95	25	46
	‘Summer green’	2	6	3.7 ± 1.1	3.8 ± 0.3	1.03	6	31

The source codes represent the following companies: 1—Mr. Fothergill’s (South Windsor, NSW, Australia); 2—Yates (Wetherill Park, NSW, Australia); 3—Green Harvest (Maleny, QLD, Australia); 4—Eden Seed (Lower Beechmont, QLD, Australia); 5—Select Organic Seeds (Lower Beechmont, QLD, Australia).

**Table 2:** Phi thickening induction by salt in different *Brassica* species and cultivars.

Species/Subspecies	Common Name	Cultivar	Source Code	Measurement Day	Root Length (cm)		Root Length Ratio	Phi Thickening Score	
					0 mM	80 mM	(80 mM/0 mM)	0 mM	80 mM
*Brassica napus*	Canola	‘Hyola97CL’	7	5	3.6 ± 0.8	2.5 ± 1.0	0.69	0	41
	Canola	‘Hyola474CL’	7	5	2.7 ± 0.6	2.1 ± 1.1	0.78	0	25
	Canola	‘Archer’	7	5	2.5 ± 0.8	2.7 ± 0.8	1.08	0	68
	Canola	‘Edimax’	7	5	2.6 ± 0.6	2.0 ± 0.9	0.77	0	8
	Canola	‘Sensation’	7	5	4.0 ± 2.5	2.3 ± 1.2	0.58	0	0
*Brassica rapa*									
subsp. *pekinesis*		‘Wong bok’	4	5	5.8 ± 1.0	4.5 ± 0.4	0.77	4	0
subsp. *pekinesis*		‘Michihli’	4	10	5.1 ± 0.7	4.8 ± 0.3	0.95	0	0
subsp. *chinensis*		‘Pak choi green’	4	5	3.4 ± 0.7	3.7 ± 0.8	1.11	0	0
subsp. *chinensis*		‘Granat’	5	6	3.8 ± 1.5	5.2 ± 0.4	1.36	0	0
subsp. *perviridis*	Salad greens	‘Komatsuna’	4	5	4.4 ± 0.8	4.5 ± 0.8	1.02	0	0
var. *japonica*		‘Mibuna’	4	8	3.6 ± 1.0	3.8 ± 1.3	1.07	0	0
subsp. *narinosa*	Salad greens	‘Tatsoi’	4	8	4.7 ± 0.9	3.8 ± 0.8	0.80	0	0
subsp. *rapa*	Turnip	‘Gold ball’	4	5	4.6 ± 0.8	4.6 ± 1.3	1.00	0	0
*Brassica juncea*	Mustard	‘Korean red’	8	6	4.0 ± 0.6	4.3 ± 0.6	1.08	0	0
	Mustard	‘Asian green’	8	6	2.0 ± 0.3	2.7 ± 0.8	1.35	0	0
	Mustard	‘broad leaf’	8	6	3.9 ± 0.3	3.3 ± 0.5	0.85	0	0
	Mustard	‘gai choy’	8	6	4.6 ± 0.4	2.8 ± 0.3	0.61	0	0
	Mustard	‘Large leaf’	9	6	1.9 ± 0.7	2.1 ± 0.7	1.11	0	0
*Brassica nigra*	Black mustard		6	5	7.3 ± 1.1	5.8 ± 0.7	0.79	0	0
	English mustard		9	6	5.5 ± 0.5	4.9 ± 0.5	0.89	0	0
*Brassica carinata*	Mustard	‘Texsel greens’	8	6	4.2 ± 0.6	3.7 ± 1.0	0.88	0	0
	Ethiopian Cabbage		3	6	4.1 ± 0.3	4.1 ± 0.6	1.00	0	0

The source codes represent the following companies and laboratories: 3—Green Harvest; 4—Eden Seed; 5—Select Organic; 6—The Seed Collection (Upper Ferntree Gully, VIC, Australia); 7—Dr Susan Sprague, CSIRO Agriculture, (Canberra, ACT, Australia); 8—4Seasons Seeds (Tenterfield, NSW, Australia); 9—Fair Dinkum Seeds (Gin Gin, QLD, Australia).

**Table 3:** Phi thickening induction by salt in different species of the Brassicaceae family.

Species	Common Name	Cultivar	Source Code	Measurement Day	Root Length (cm)		Root Length Ratio	Phi Thickening Score	
					0 mM	80 mM	(80 mM/0 mM)	0 mM	80 mM
*Sinapsis alba*	White mustard		1	5	2.8 ± 0.9	2.4 ± 1.1	0.84	102	95
*Iberis amara*	Candytuft	‘Iceberg’	6	8	3.2 ± 1.7	2.2	0.67	22	46
*Lobularia maritima*	Alyssum	‘Rosie O’Day’	4	6	1.5 ± 0.2	2.0 ± 0.4	1.37	15	43
*Thlaspsi cauelescens*	Alpine pennycress		13	11	3.1 ± 0.4	1.8 ± 0.3	0.58	15	16
*Malcolmia maritima*	Stock	‘Early giant imperial’	4	6	3.6 ± 0.6	2.5 ± 0.9	0.69	0	0
*Herperis matronalis*	Dame’s white rocket		10	13	2.0 ± 0.8	2.3 ± 0.6	1.16	0	0
*Diplotaxis erucoides*	White Rocket	‘Wasabi’	10	5	2.0 ± 0.4	1.9 ± 0.4	0.99	0	0
*Diplotaxis muralis*	Wild rocket	‘Salad greens’	4	11	1.5 ± 0.1	1.97	1.36	0	0
*Eruca sativa*	Rocket		4	5	2.5 ± 0.6	2.5 ± 0.8	1.00	0	0
*Eruca vesicaria*	Arugula		8	5	2.7 ± 1.1	4.5 ± 1.3	1.67	0	0
*Isatis tinctoria*	Dyers woad		10	6	2.4 ± 0.6	2.2 ± 0.9	0.93	0	0
*Cheiranthus cheiri*	Wallflower	‘Fire king’	6	5	3.5 ± 0.6	2.7 ± 0.8	0.77	0	0
*Matthiola incana*	Virginian stock		1	8	3.4 ± 1.3	2.9 ± 0.3	0.86	0	0
*Aubrieta* sp.	Rock cress		11	11	1.8 ± 0.8	0.21	0.12	0	0
*Lepidium sativum*	Plain cress		5	5	5.5 ± 1.3	5.2 ± 0.9	0.93	0	0
*Cardamine hirsuta*	Hairy bittercress		12	11	1.7 ± 0.8	1.7± 0.0	1.00	0	0
*Arapidopsis thaliana*		Ecotype Col-0	14	14	1.9 ± 0.7	2.8 ± 0.7	1.47	0	0
*Nasturtium officinale*	Watercress	‘Aqua large leaf’	3	11	1.6	0.53	0.33	0	0
*Barbarea verna*	America upland cress		4	35	1.1	0.9	0.81	0	0
*Crambe maritima*	Sea kale		8	35	3.8	-	-	0	-
*Lunaria biennis*	Money plant		10	13	3.9 ± 1.0	-	-	0	-

The source codes represent the following companies and laboratories: 1—Mr. Fothergill’s; 3—Green Harvest; 4—Eden Seed; 5—Select Organic; 6—The Seed Collection; 8—4Seasons Seeds (Tenterfield, NSW, Australia); 10—Southern Harvest (Kingston, TAS, Australia); 11—Boondie Seeds (Armidale, NSW, Australia); 12—Dr John Bowman (Monash University, VIC, Australia); 13—Dr Damien Callahan (Deakin University, VIC, Australia); 14—Arabidopsis Biological Resource Center.

## Supplementary Materials

The following are available online at http://www.mdpi.com/2223-7747/7/2/47/s1, Figure S1: Computer-generated re-slices through optical stacks collected from roots mounted longitudinally showing berberine-stained lignin (green in overlay) and pontamine-stained cellulose (red in overlay) in *B. napus* ‘Hyola971CL’ roots. (a) ‘Hyda971CL’ grown on control plates (no NaCl). Casparian strips (Cs) weakly labelled with berberine in the endodermis while xylem (x) strongly labelled in the central vascular tissue. (b) ‘Hyda971CL’ 5 day-old root treated with 80 mM NaCl induced formation of phi thickenings (Φ) in the innermost cortex immediately outside the endodermis containing Casparian strip (Cs). Bar = 100 μm. Movie S1: 3D reconstructions of confocal optical stacks of berberine-stained lignin (green) and pontamine-stained cellulose (red) in *B. oleracea* roots of cultivar ‘Marathon F1’. Control root in the absence of added salt, which weakly labelled the Casparian strips in the endodermal layer and strongly labelled the xylem bundles. Movie S2: 3D reconstructions of confocal optical stacks of berberine-stained lignin (green) and pontamine-stained cellulose (red) in *B. oleracea* roots of cultivar ‘Marathon F1’. Root grown in presence of 80 mM NaCl which strongly induced the formation of phi thickenings.
