# Supplementary figures and images for: Developmental Biology and Induction of Phi Thickenings by Abiotic Stress in Roots of the Brassicaceae
Source: Plants (Basel). 2018 Jun 19;7(2):47. doi: 10.3390/plants7020047 (PMC6027303; doi:10.3390/plants7020047)

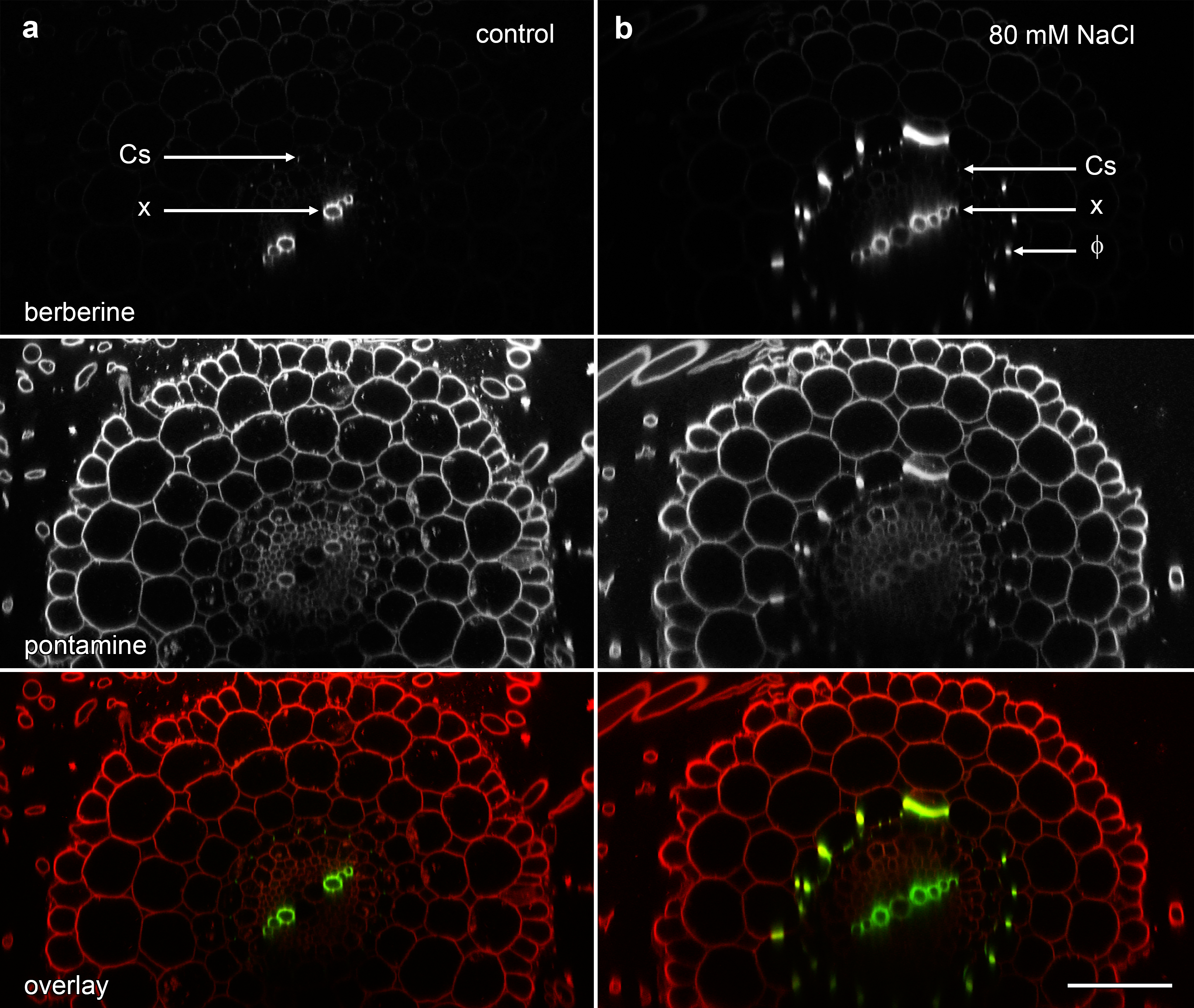

Supplement: Supplementary file 1 [file plants-07-00047-s001.zip › Supplementary Figure 1.tif]
